# The effect of Photobiomodulation therapy on implant stability in patients receiving dental implants- a systematic review and meta-analysis

**DOI:** 10.1186/s12903-025-06253-2

**Published:** 2025-07-02

**Authors:** Ashika Singhania, Anjali Borle, Surekha Godbole, Seema Sathe

**Affiliations:** 1 Datta Meghe Institute of Higher Education and Research, Wardha, India; 2 Wardha, India

**Keywords:** Photobiomodulation, Implant stability, Low-level laser, Implant dentistry, CBCT, Radiographs

## Abstract

**Background:**

The use of photobiomodulation (PBM) therapy in implant dentistry for enhancing the osseointegration is proven in many studies. However, the cumulative data of its effect around dental implants in patients is limited.

**Objectives:**

The purpose of the present study was to evaluate the effect of the PBM therapy on primary and secondary implant stability.

**Material and methods:**

The studies included in the review and meta-analysis were selected according to the Preferred Reporting Items for Systematic Reviews and Meta-Analyses (PRISMA) guidelines and the PICOS criteria. The RoB 2 tool was used for assessing the risk of bias and the RevMan software, v. 5.4, was used for meta-analysis. Quantitative analysis was done using two tools considering the implant stability measurement using Ostellmeter and radiographs as the outcome. The mean and standard deviation (M ± SD) values for implant stability as well as the sample size were extracted from the articles, and the inverse variance method with random effects was used for meta-analysis. The forest plots for all time intervals were inspected to estimate the heterogeneity.

**Results:**

A total of 117 articles were initially retrieved, out of which 62 remained after duplicate removal. 15 articles were included in the review for qualitative analysis and 8 were eligible for quantitative analysis. The meta-analysis showed that at 2 weeks, 4 weeks and 8 weeks, the control group showed better result than experimental group, at 3 weeks the implant stability favoured the experimental group whereas at 6 and 12 weeks there was no difference seen between control and experimental group.

**Conclusions:**

The PBM therapy on implant stability has yielded mixed results. While some studies have demonstrated a positive effect, others have reported no significant impact. Consequently, further research is warranted to fully elucidate the effects of PBM therapy on implant stability.

## Introduction

Humans have always had to deal with tooth loss, and in the modern world, as life expectancy has increased, so has the impact of age on tooth loss. For people of all ages, attempts to replace lost teeth have proven essential, and Branemark has accomplished this by developing endosseous dental implants. He identified the phenomenon known as osseointegration—the tight bond between titanium implants and bone that is strong enough to transfer force [1]. According to biological theory, osseointegration is an immune-driven process that produces new bone and for protecting the implant from the surrounding tissues, the immune system first perceives the implant as a foreign body and then builds bone around it [2]. Preliminary guidelines indicate that 3–4 months will pass after implant insertion for healing. Because there is more cancellous bone in the maxilla and posterior mandible, this process takes longer and could take up to six months. The key requirement for implant loading is that the implant must have sufficient primary stability at the moment of implantation. Dental implants'primary stability is influenced by a number of parameters, including as surface topography, implant morphology, bone quantity and quality, and surgical technique [3]. Both the surgical approach and the characteristics of the dental implant affect secondary stability. However, secondary stability is also directly impacted by primary stability. Determining the optimal loading time thus requires evaluating implant stability at various time periods [4].

Implants frequently lack the ideal circumstances for both the amount and quality of bone. Photobiomodulation, or low-energy photon irradiation by light in the far-red to near-infrared (NIR) spectrum (630–1000 nm) utilizing low-energy lasers or light-emitting diodes (LED), is one of the potential techniques to promote osseointegration. When cytochrome c oxidase, a photoacceptor molecule in the mitochondria, absorbs light, it changes the cellular process and increases the generation of adenosine triphosphate (ATP), which promotes wound healing in injured and ischemic tissues [5]. This light source is necessary for the activation of the light sensitive substance (PS) by exposing a low power light at visible wavelengths and activating it. Additionally, it promotes osteoblast development and proliferation, which biostimulates bone tissue. PBM has been investigated for its possible use in implant dentistry to enhance implant stability and the general healing of the hard and soft tissues surrounding dental implants. LLLT has been shown to have a beneficial effect around the implant in a number of in vitro and animal investigations [6]. This systematic review and meta-analysis were conducted on the basis of clinical studies to evaluate the qualitative and quantitative effect of PBM on implant stability.

## Methods

This systematic review and meta-analysis were performed according to the Preferred Reporting Items for Systematic Reviews and Meta-Analyses (PRISMA) guidelines [7]. The review protocol was registered under PROSPERO International Prospective Register of Systematic Reviews (CRD42024427943).

### Study design

Randomised controlled trials(RCT’s) on humans.

### Search strategy

The search strategy was based on the PICO criteria due to the comparative nature of the study: Population(P): male or female patients requiring the replacement of one or more missing teeth with dental implants, Intervention(I): the PBM therapy around dental implants, Comparison(C): patients who did not receive PBM or any other adjunctive therapy around dental implants, Outcome(O): the effect of the PBM therapy on primary and secondary implant stability. An advanced search of electronic databases was carried out by 2 investigators individually without consulting one another. The following terms were searched in all data bases, in Pubmed and Cochrane the search strategy followed was as follows. For population following search terms were used"low intensity laser therapy"[Title/Abstract] OR"low intensity laser therapy"[Title/Abstract] OR"low level laser therapy"[Title/Abstract] OR"low level laser therapy"[Title/Abstract] OR"photobiomodulation*"[Title/Abstract] OR"Photobiomodulation therapy"[Title/Abstract] OR"low level light therapy"[Title/Abstract] OR"low level light therapy"[Title/Abstract] OR"low level light therapy"[MeSH Terms]. For intervention following search terms were used"dental implant*"[Title/Abstract] OR"implant*"[Title/Abstract] OR"dental implantation"[MeSH Terms]. In The electronic databases were searched for articles published till January 2024. To avoid missing any eligible studies, the references of all eligible articles were also searched. The grey literature was also searched using Proquest and Google scholar and no relevant data was found. The data of 117 articles was uploaded on Rayyan where two blinded investigators screened the abstracts, removed 55 duplicates, title and abstract screening was done for 62 articles. Later eligible articles fulfilling the inclusion criteria were further assessed by accessing their full text from relevant sources by 2 investigators individually. Any discrepancy between 2 investigators was resolved by a third investigator. The qualitative analysis was done for 15 articles and quantitative analysis was done for 8 articles.

### Inclusion and exclusion criteria

This review focussed on randomised controlled trials (RCT) or quasi RCT on humans reporting the effects of Photobiomodulation on primary and secondary implant stability assessed using Ostellmeter in ISQ scale and Radiographs. RCT that used LED as photobiomodulation therapy was excluded, also case reports, review papers, clinical trials, letters to editors, monographs, in vitro studies, animal studies.

### Data collection and analysis

The following information was extracted from the studies included in this review by 2 independent reviewers: first author(year),country, sample size, study design, mean age of patient, implant site, type of implant, outcome, evaluation time, study conclusion. Another data extraction was done for the laser parameters which included: Study, type of LLLT, wavelength, mode, output/energy density, irradiation time and energy per point and number of points, total energy per session, frequency of laser treatment.

Statistical analysis was conducted using RevMan version 5.4 provided by Cochrane collaboration [7]. The meta‑analysis was conducted using the fixed‑ or random‑effects methods. Fixed‑effects meta‑analysis was used when the heterogeneity was small (I2 < 50%, *P* < 0.05). When the heterogeneity was large (I2 > 50%, *P* > 0.05), a random‑effects model analysis was undertaken considering 95% confidence interval. If a meta-analysis could not be performed, data was summarized qualitatively.

### Risk of bias assessment

The risk of bias of all the studies included in the review was assessed by two investigators who were blinded, using the Cochrane risk of bias assessment tool [8]. The agreement between reviewers was assessed based on Cohen’s kappa statistics, assuming k = 0.6 to be an eligible score [9]. According to ROB 2 tool, 5 domains were taken into consideration to evaluate each article in terms of risk of bias, namely randomisation process, deviation from intended interventions, missing outcome data, measurement of outcome, selection of the reported results. Accordingly the risk of bias of particular studies was calculated as high, low or unclear [10].

## Results

**Fig. 1:**
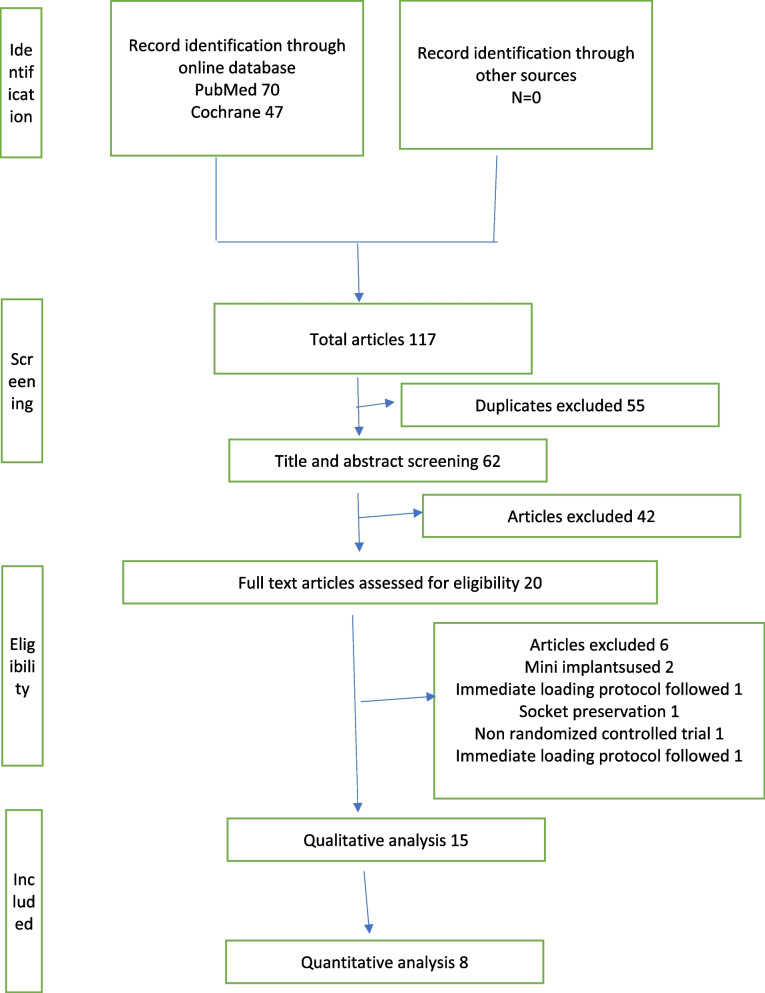
Flowchart depicting the study selection process

During the search process, a total of 117 articles were identified (Fig. 1) from electronic databases, out of which 62 articles remained after removal of 55 duplicates. 42 articles were excluded based on exclusion criteria and at the end of study selection, 15 articles were selected for qualitative analysis [1, 3, 5, 13–24] and 8 articles for quantitative analysis [2, 11–15, 19, 21].The inter-investigator reliability in the selection of articles was statistically evaluated using Cohen’s kappa analysis and a score of 0.97 was obtained.

The risk of bias in the study included in the review was assessed according to the ROB2 tool (Table 1). Out of 15 studies, 6 studies showed high risk of bias [1, 4, 16–18, 20, 22], 1 study showed some concerns [11]and 7 studies were low risk of bias [2, 12–15, 19, 21].In the high risk studies, 2 studies had missing outcome data (D3) [1, 4, 22], studies had different outcome measurement(D4) 16–18, 20] and 2 studies did not adhere with randomisation process (D1) [1, 4].

**Table 1:**
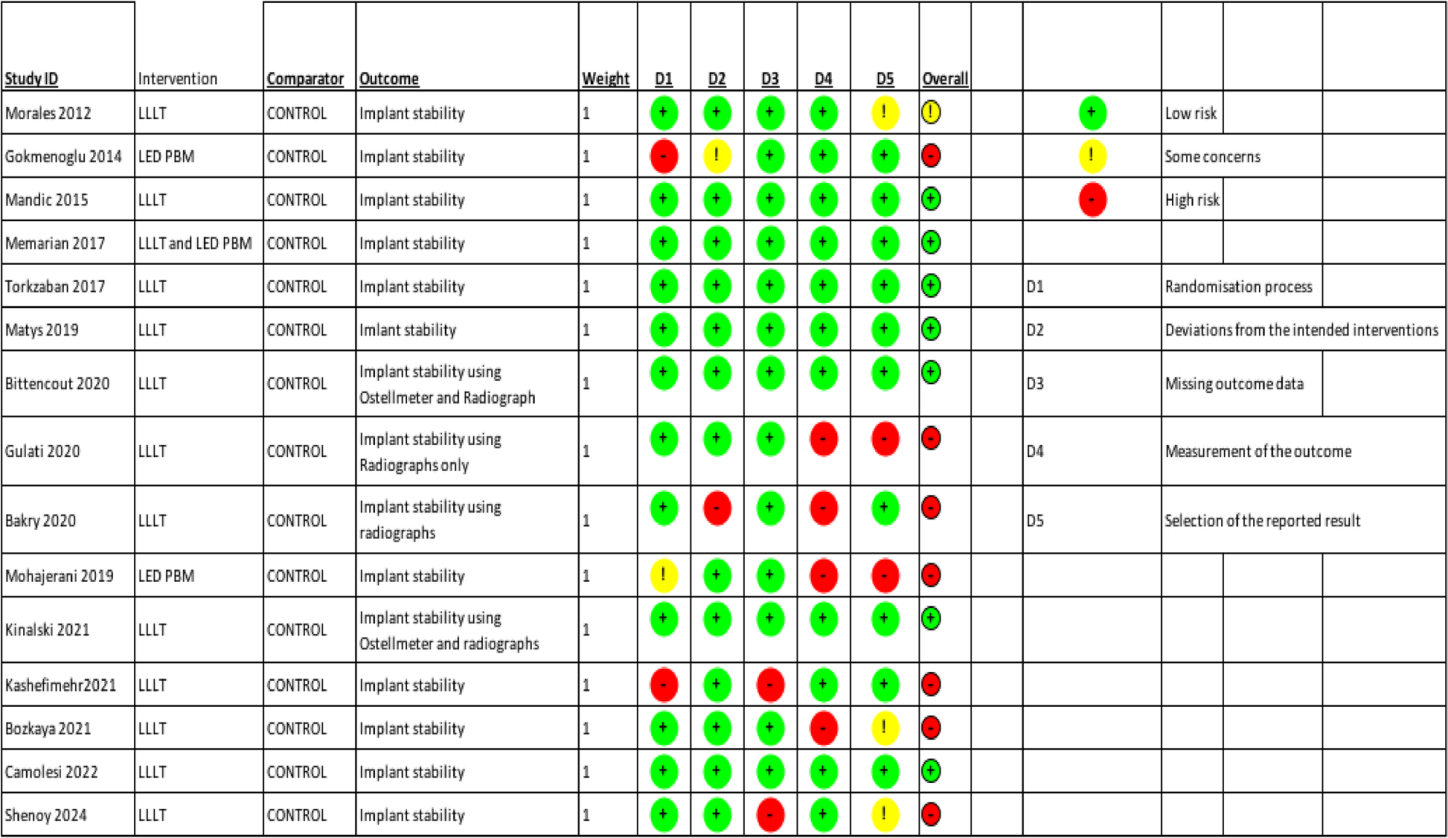
Risk of bias assessment of the included studies with the use of the RoB 2 tool

### Characteristics of systematic review

All the articles included in the systematic review were randomised controlled trials, and were published between 2012 and 2024. Six studies had a split mouth design [1, 11–13, 20, 22 ] whereas 9 studies had a case control design [2, 4, 14–19, 21]. The studies were conducted in Brazil, Turkey, Serbia, Iran, Poland, India, Egypt, Spain. In 5 studies implant placement was done in mandible [11, 13, 14, 16, 18] in 3 studies implant was placed in maxilla [2, 12, 17] and in 3 studies bilateral mandibular or maxillary implants [1, 20, 22] was placed and implant site was not mentioned in 4 studies. The primary outcome, implant stability was assessed in ISQ scale in 11 studies, in 2 studies PTV value was assessed. The implant stability was assessed using Ostellmeter in majority of studies. In 2 studies implant stability was assessed radiographically, Gulati et al. assessed using IOPA and CBCT and Bakry et al. assessed using RVG. In study by Mandic et al., Alkaline phosphatase level was also assessed, Memarian et al. assessed GCF fluid for levels of IL‐1β and PGE2 markers, Bakry et al. assessed the probing depth, Bozkaya et al. assessed the microbiological changes, Camolesi et al. assessed the post-surgical healing and inflammation. In the CBCT analysis by Matys et al., the bone density level(grayscale value) was measured at three levels cervical, middle and apical and in the CBCT analysis by Gulati et al., average crestal bone level value was made on four anatomical sites mesial, distal, buccal and lingual. In IOPA analysis by Bittencout et al., Bakry et al. and Kinalski et al. implant platform to bone crest measurement at insertion time and abutment stage was recorded whereas Gulati et al. did IOPA assessment in mesial and distal site. The evaluation period was different for all the studies and varied from baseline to 1 year (Table 2).

**Table 2:** Characteristics of included studies

Author(Year)	Country	Sample size	Study design	Mean age	Implant site	Type of implant	Bone type	Outcome	Evaluation time	Study conclusion
Morales et al. 2012	Brazil	LG 15 CG 14	Split mouth	36	Posterior mandible	Dentsply, Germany with diameter of 3.8 mm and a length of 11 mm		Implant stability in ISQ scale	baseline, 10 days, 3, 6, 9, and 12 weeks	no effect
Gokemenoglu 2014	Turkey	LG 10 CG 12	Case control	48		Dentsply, Manheim, Germany	Type 2 or 3	Implant stability in ISQ scale	immediately, 2, 4, 8 and 12 weeks postoperatively	positive effect
Mandic 2015 [12]	Serbia	LG 20 CG 20	Split mouth	61	posterior maxilla	Bredent, Senden, Germany		Implant stability in ISQ scale, Alkaline phosphatase level and early implant success rate	**Implant stability:** immediately and 1, 2, 3, 4, 5, 6 weeks postoperatively **PICF:** postoperative, 7, 14, 21, 28 days **Early implant success rate:** 6 weeks postoperatively	no effect
Memarian 2018 [13]	Iran	LG 12 LG 12 CG 12	Split mouth		anterior mandible	Dio, Busan, South Korea	Type 2 or 3	implant stability (PTV) and GCF fluid for levels of IL‐1β and PGE2 markers	implant stability: day of surgery, 3, 4, and 8 weeks after surgery Inflammatory markers: 4 and 8 weeks postoperatively	positive effect
Torkzaban 2018	Iran	LG 40 CG 40	Case control	42	maxilla	Dio, Busan, South Korea	D3, D4	Implant stability in ISQ scale	immediately, 10 days, 3, 6 and 12 weeks postoperatively	no effect
Matys 2019 [14]	Poland	LG 18 CG 22	Case control	47	posterior mandible	Dentium, cypress, USA	D2	implant stability(PTV) and CBCT	PTV: after surgery, 2 weeks, 4 weeks, 2 months and 3 months after surgery CBCT: after surgery, 4 weeks and 3 months after surgery	positive effect
Bittencount 2019	Brazil	LG 23 CG 23	Case control	51		Straumann, Basel, Switzerland		Implant stability in ISQ scale and IOPA	during implant placement and at abutment selection phase	no effect
Gulati 2020 [16]	India	LG 10 CG 10	Case control	35	posterior mandible	Adin Dental, Poland	type 2 or 3	implant stability using IOPA and CBCT	IOPA: immediately, 6 weeks, 6 months and 1 year following prosthesis loading CBCT: immediately and at 1 year	positive effect
Bakry 2020 [17]	Egypt	LG 6 CG 6	Case control	40	posterior maxilla	Zimmer Dental, Carlsbad, USA		crestal bone level using RVG, clinical parameters probing depth	baseline, 2 weeks, 3 months, and 6 months	no effect
Mohajerani 2020	Iran	LG 28 CG 28	Case control	38	posterior mandible	Zimmer Dental, Carlsbad, USA		Implant stability in ISQ scale	immeditaley, 10, 21, 42 and 63 days postoperatively	positive effect
Kinalski 2021 [19]	Brazil	LG 32 CG 32	Case control	49.94		Alvim CM, Neodent Straumann, Curitiba, Brazil		Implant stability in ISQ scale and Digital radiographs	baseline, at sbutment selection phase(4–6 months)	no effect
Kashefimehr 2021 [1]	Iran	LG 12 CG 12	split mouth	44.75	bilateral mandibular or maxillary implants	Dio, Busan, South Korea	Type 3 or 4	Implant stability in ISQ scale	immediately, one month and 3 months	positive effect
Bozkaya 2021 [20]	Turkey	LG 47 CG 46	split mouth	55	bilateral mandibular or maxillary implants	Straumann Stan- dard Plus Implant; Institute Straumann AG, Waldenburg, Switzerland)		Implant stability in ISQ scale and microbiological changes	baseline, 30, 60 and 90 days	no effect
Camolesi 2022	Spain	LG 20 CG 20	Case control			Model IPX, Nueva Galimplant	Type 1, 2, 3, 4	Implant stability in ISQ scale Post-surgical healing and inflammation	immediately, 7 days, 4 weeks, 8 weeks	no effect
Shenoy 2024 [22]	India	LG 20 CG 20	split mouth	18–65	bilateral mandibular or maxillary implants	Osstem implant TS III SA, Osstem Implant Co., Seoul, Korea		Implant stability in ISQ scale	1, 2, 4 and 12 weeks postoperatively	positive effect

The type of laser and protocol used for PBM is presented (Table 3). Diode lasers used in all the studies varied from 626-980nm. The time of laser application varied in all the studies ranging from before placement to 24 days after implant placement.

**Table 3:** Laser specifications

Study	Type of LLLT	Wavelength	Mode	Output/Energy density	irradiation time and energy per point and number of points	total energy per session	frequency of laser treatment
Morales 2012	GaAIAs laser	830 nm	contact continuous	86 ± 2 mW, 92.1 J/cm2	**irradiation time per point**: 3 s **total points:** 20 (9 at the vestibular, 9 at the lingual, 1 at mesial and 1 at distal) **energy per point:** 0.25 J per point, 5 J per implant	8 J	immediately, 2, 4, 8,10, 12 and 14 days
Gokemenoglu 2014	LED(Osseopulse Ar 300)	626 nm	NR	100 mW, 28.37 J/cm2	**irradiation time per point**: 20 min **total points:** 2 (buccal and lingual) **energy per point:** 20 J/cm2	222 J	3 times per week for 3 weeks
Mandic 2015 [12]	GaAIAs laser	637 nm	contact continuous	40 mW, 6.26 J/cm2			immediately after surgery, 1, 2, 3, 4, 5, 6, 7 days later
Memarian 2018 [13]	Diode laser	Laser: 810 nm LED: 626 nm	contact continuous	Laser: 50 mW LED: 185 mW	**irradiation time per point**: Laser 400 s, LED 20 min **total points:** 2 (buccal and lingual) **energy per point:** 20 J/cm2,	222 J	day of surgery, 3, 7, 10 and 14 days
Torkzaban 2018	Biolase diode laser	940 nm	contact continuous	100 mW, 354.6 J/cm2	**irradiation time per point**: 40 s **total points:** 2 (buccal and lingual) **energy per point:** 4 J	56 J	immediately, 2, 4, 6, 8, 10 and 12 days after insertion
Matys 2019 [14]	red diode laser	635 nm	contact continuous	100 mW, 99.04 J/cm2	**irradiation time per point**: 40 s **total points:** 2 (buccal and lingual) **energy per point:** 4 J	56 J	1 day before surgery, immediately after surgery, 2, 4, 7 and 14 days later
Bittencount 2019	GaAIAs laser	808 nm	contact continuous	50 mW	**irradiation time per point**: 1.23 min **total points: 6** (2 buccal, 2 lingual and 2 occlusal) **energy per point:** 11 J	66 J	before implant placement after suturing
Gulati 2020 [16]	GaAIAs laser	980 nm	contact continuous	0.1 W	**irradiation time per point**: 1.23 min **total points: 6** (mesiobuccal, distobuccal, midbuccal, mislingua, mesial, distal) **energy per point:** 1 J	6 J	after implant placement, 3, 7 and 14 day
Bakry 2020 [17]	smart M lasotronix Pro diode laser	980 nm	contact continuous	2 W	**irradiation time per point**: 5 min **total points:** buccal, lingual as well crestal surfaces		two weeks before implant placement, 2, 4, 6, 8, 10, 12, 14, 16, 18, 20, 22, 24 days postoperatively
Mohajerani 2020	LLLT and LED device	830 nm laser with 632 nm LED	contact continuous	Laser 15 mW/cmsq LED 10 mW/cmsq	**irradiation time per point**: 20 min **total points:**four points around the implants		Everyday for 10 days
Kinalski 2021 [19]	GaAIAs laser	808 nm	contact continuous	50 mW	**irradiation time per point**: 80 secs **total points:** 6 points in the labial region where the implant would be placed (apical and cervical): two points in the lingual region (apical and cervical) and two points in the occlusal direction **energy per point:** 1 J	66 J	Before implant placement after suturing
Kashefimehr 2021 [1]	LED(Osseopulse Ar 300)	626 nm			**irradiation time per point**: 20 min		1 days before surgery, 10 days consecutive sessions including day of surgery
Bozkaya 2021 [20]	GaAIAs laser	830 nm	non contact continuous with application distance 0.5 to 1 cm	300 mW	**irradiation time per point**: 3 s **total points:** 20 points in the eight points at the vestibular, eight points at the lingual, two at the distal, and two at the mesial region of the implant **energy per point:** 0.3 J	6 J	immediately and 3 times a week for 15 days
Camolesi 2022	GaAIAs laser	630 nm and 808 nm	contact continuous	100 mW	**irradiation time per point**: 100 s **total points: T**hree points were irradiated: the buccal side (4 J) and the palatal or lingual side (4 J) using the infrared light mode (808 nm) and the occlusal side (2 J) using the red- light mode (630 nm) **energy per point:** 0.1 J	10 J	immediately and after 7 days
Shenoy 2024 [22]	GaAIAs laser	940 nm	contact continuous	100 mW	**irradiation time per point**: 40 s **total points:** 2 (buccal and lingual) **energy per point:** 14.18 J/cmsq	8 J	immediately, 2, 4, 6, 8, 10 and 12 days after insertion

### Meta-analysis

A meta-analysis was performed to evaluate the effect of PBM therapy on implant stability measured in ISQ or RFA scale and using Radiographs. The analysis was divided into two outcomes and each outcome was divided into subgroups based on evaluation time of the implant stability. At 2 weeks, 4 weeks and 8 weeks, the control group demonstrated significantly higher implant stability compared to the experimental group and statistically significant result (*p* = 0.0001, *p* = 0.0006, *p* = 0.04). There was low to moderate heterogeneity seen with I^2^ = 23% at 8 weeks, I^2^ = 50% at 4 weeks and I^2^ = 64% at 2 weeks. At 3 weeks, the implant stability significantly favoured the experimental group with *p* < 0.00001 but the heterogeneity being high I^2^ = 98%. At 6 weeks and 12 weeks, there was no statistical significant difference seen between the groups (*p* = 0.67, *p* = 0.45) and heterogeneity being I^2^ = 1% at 6 weeks and I^2^ = 64% at 12 weeks (Table 4).

**Table 4:**
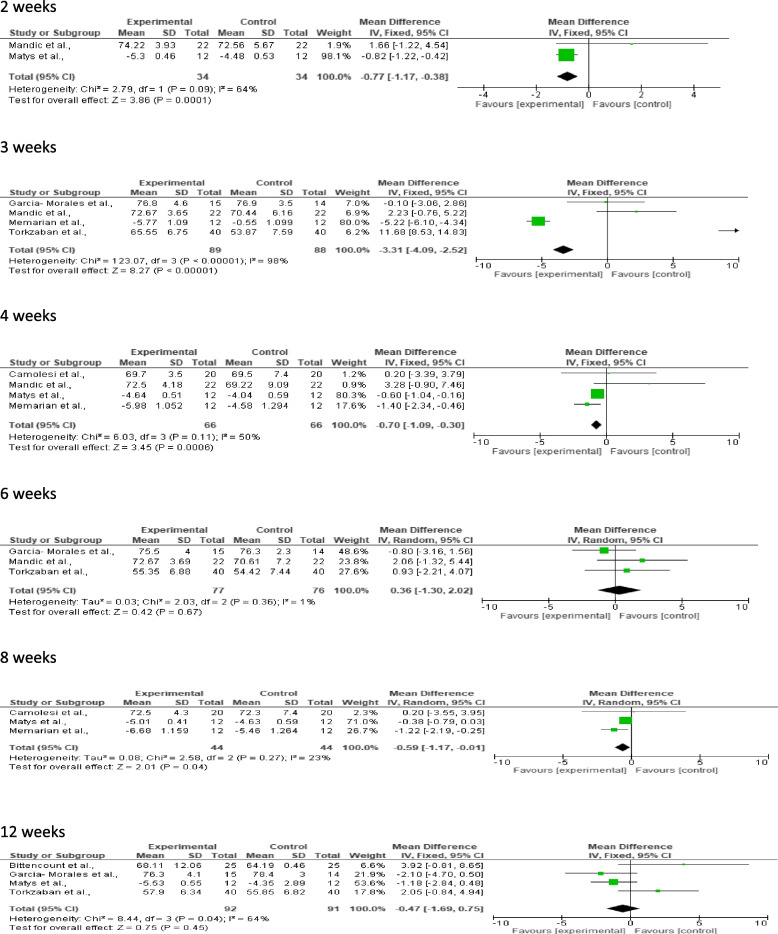
Forest plots of dental stability for experimental (laser) and control groups at 2 weeks, 3 weeks, 4 weeks, 6 weeks, 8 weeks, and 12 weeks

In the second outcome, where the implant stability was assessed using radiographs. The overall result and heterogeneity of implant platform to bone crest at insertion time was statistically not significant (*p* = 0.45, *p* = 0.30 I^2^ = 8%). The overall result was statistically non-significant and heterogeneity of implant platform to bone crest at abutment stage was statistically significant (*p* = 0.90, *p* = 0.02 I^2^ = 57%). The overall pooled mean difference between the groups was 0.17 with 95% CI (−0.39, 0.73) which was found to be not significant statistically (*p* = 0.86) (Table 5).

**Table 5:**
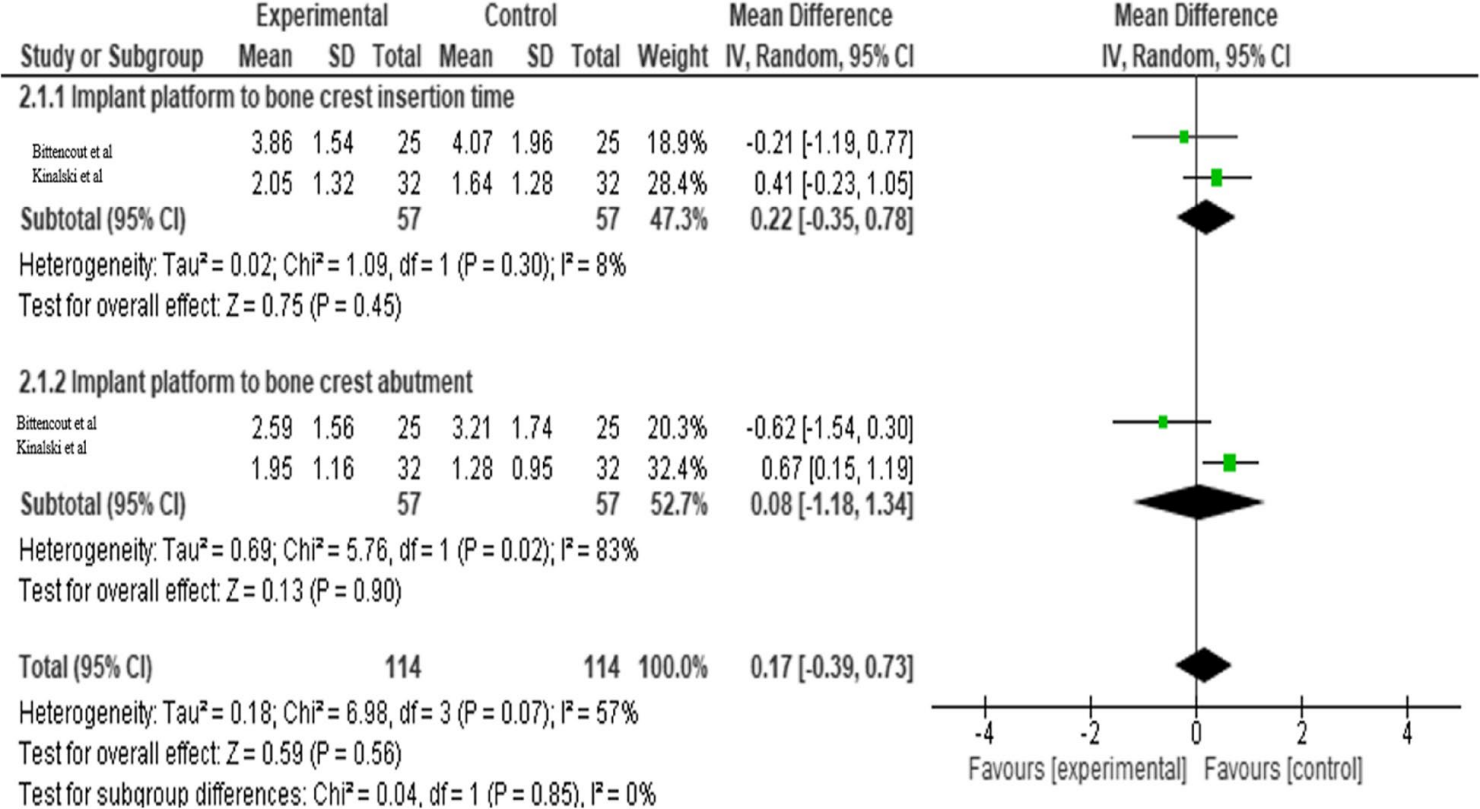
Forest plots of dental stability using IOPA for experimental (laser) and control groups at insertion time and at abutment time

## Discussion

There are not many systematic reviews and meta-analyses conducted evaluating the effect of PBM therapy on implant stability and also the implant stability assessed using radiographs is not assessed in these meta-analyses. This review was conducted to evaluate the effect of PBM therapy on implant stability. Out of various techniques to assess the implant stability, the RFA technique is routinely used in clinical practice because it is easy to perform and also there is strong correlation between RFA and histomorphometric measures. Other than this insertion torque values, reverse torque test, percussion test, CBCT, radiography, and histomorphometric analysis are some of the techniques utilized to evaluate the stability of the implant. Among these the most reliable are ISQ, PTV and meta-analysis is conducted for same but digital assessment through CBCT and radiographs are also included in these meta-analyses. Out of all the studies assessed for meta-analyses, positive effect was seen in 2 studies [13, 14] and other studies showed no significant difference or effect. Another meta-analysis conducted by Remisio et al. which included all preclinical studies where assessment of osseointegration was done. There was no statistical difference between studies at the 2 month follow-up (*p* = 0.672) but this difference was significant at 1 and 3 months (*p* < 0.001) [25]. Similarly in the present meta-analysis, the studies included for qualitative analyses and not for quantitative analysis, 5 studies showed positive effect [1, 4, 16, 18, 22].

The studies by Gokmenoglu et al., Mohajerani et al. [4, 18] used LED for their intervention group, although it gave a positive effect but other studies gave intervention only through PBM therapy. Using the same wavelength of 980 nm, Gulati et al. and Bakry et al. [16, 17] found different results. This could be because histological observations and gene expression analyses showed that 980 nm diode laser irradiations reduced the number of inflammatory cells and accelerated bone regeneration by increasing the number of fibroblasts and osteoblasts [26]. By evaluating the PTV value and CBCT, Matys et al. [14] shown a favorable effect between the laser and control group in their study. The data show that the stability of the bone healing process typically decreases, rises, or plateaus in the weeks that follow. After three months, the secondary stability measured in the laser group was higher than at the baseline, whereas it was lower in the control group. Torkzaban et al. and Shenoy et al. [2, 22] used the same wavelength of 940nm in their study and the irradiations given were also of the same days but Shenoy et al. showed positive effect which may be due to LLLT may vary due to individual patient factors and individual variables. Crestal bone loss was assessed by Matys et al., Bittencount et al., Gulati et al., Bakry et al. and Kinalski et al. but there was variability of assessment where only Matys et al. and Gulati et al. did the CBCT assessment and showed positive effect and IOPA analysis was done by others.

As shown in the meta-analysis statistically significant results were not observed when the overall result between the studies was assessed. This could be the result of non-homogeneity in the laser parameters—wavelength, mode, output, energy density, exposure duration, and treatment frequency—that were employed in the various experiments. Moreover, a split mouth design was used in many trials, which dispersed laser energy and could have an impact on the outcomes [1, 11–13, 20, 22]. Hence a standardised protocol for PBM therapy should be implemented.

### Limitations of study

This systematic review included 15 articles eligible for qualitative analysis, but only 8 articles met the criteria for quantitative analysis, which may limit the robustness of the findings. Notably, the baseline parameters across studies exhibited significant variability, particularly regarding evaluation time, study design, and bone type. These discrepancies resulted in inconsistent baseline parameters, which may have influenced the collected data and analysis of results, especially with regards to implant stability. Given these limitations, further research in this field is warranted, and a future review incorporating a larger, more homogeneous dataset would be valuable to provide more definitive conclusions.

### Directions for future research

There should be more high-quality RCT done in light of the aforementioned limitations. Patients'bone health and the PBMT criteria ought to be covered in full. The study's findings should be appropriately reported, and any missing data should allow for the substantial conducting of meta-analyses.

## Conclusion

Studies currently conducted do not provide a standardized procedure for PBM to improve implant stability or success rates in people. It would be very beneficial to further investigate this technology using other implant systems and characteristics, as well as varied bone states, LLLT parameters, and follow-up period durations.

## Data Availability

The datasets used and/or analysed during the current study are included in the published article and the raw data is available from the corresponding author on reasonable request.
